# Risk factors and outcomes of fetal macrosomia in a tertiary centre in Tanzania: a case-control study

**DOI:** 10.1186/s12884-016-1044-3

**Published:** 2016-08-24

**Authors:** Aisha Salim Said, Karim Premji Manji

**Affiliations:** 1 London Health Centre, PO Box 2562, Dar-es-Salaam, Tanzania; 2 Muhimbili University of Health and Allied Sciences, PO Box 65001, UN road, Dar-es-Salaam, Tanzania

## Abstract

**Background:**

Fetal macrosomia is defined as birth weight ≥4000 g. Several risk factors have been shown to be associated with fetal macrosomia. There has been an increased incidence of macrosomic babies delivered and the antecedent complications.

This study assessed the risk factors, maternal and neonatal complications of fetal macrosomia in comparison with normal birth weight neonates.

**Methods:**

A case-control study was conducted at the Muhimbili National Hospital (MNH) maternity and neonatal wards. Cases comprised of neonates with birth weight ≥4000 g; controls were matched for sex and included neonates weighing 2500–3999 g. Detailed clinical and demographic information and laboratory investigations which included blood glucose, hematocrit and plasma calcium were collected. The child was followed up to discharge/death.

**Results:**

The prevalence of macrosomic babies was 2.3 % (103 out of 4528 deliveries). Mean birth weight of macrosomic babies was 4.2 ± 0.31 kg whereas in the controls it was 3.2 ± 0.35 kg. Maternal weight ≥80 kg, maternal age ranging between 30 and 39 years, multiparity, presence of diabetes mellitus, and gestational age ≥40 years, previous history of fetal macrosomia and delivery weight ≥80 kg were significantly associated with fetal macrosomia. Macrosomic infants were more likely to have birth asphyxia, shoulder dystocia, hypoglycemia, respiratory distress and perinatal trauma and increased risk of death compared to controls. Maternal complications such as postpartum hemorrhage, second degree perineal tears and prolonged labor occurred more frequently in the macrosomia group compared to controls (*p*-value <0.05), while shoulder dystocia, uterine rupture and maternal death were recorded only among the cases and none occurred in the controls.

**Conclusion:**

Fetal macrosomia was an important cause of maternal and neonatal morbidity at Muhimbili National Hospital. Presence of risk factors should alert the obstetrician to closely monitor these pregnancies and plan on appropriate mode of delivery. Macrosomic neonates should be routinely screened and appropriately managed for hypoglycemia.

## Background

Fetal macrosomia is common in obstetrics with problems to both the mother and the newborn. It has been associated with significant risk of morbidity and mortality. Over the years, the trend in fetal macrosomia has been shown to be increasing worldwide [1–3].

Several risk factors have been identified in the causation of macrosomia. These include maternal diabetes, high pre-pregnancy Body Mass Index (BMI), excessive weight gain during pregnancy, multiparity, male sex, parental height, and prolonged gestation [3–18].

During labour, cephalo-pelvic disproportion can result in fetal distress and difficult deliveries are frequent in this group of infants. Maternal complications include increased risk of Caesarian section, postpartum hemorrhage and perineal lacerations [3, 8, 19]. The risk increases with a higher birth weight of the infant [20].

Neonatal complications include birth asphyxia, birth trauma, and hypoglycemia [8, 19, 21–25]. Polycythemia and hypocalcemia are most often noted in infants born to diabetic mothers. Furthermore, these infants may be at a higher risk of obesity and diabetes later in life [26, 27].

The few studies on macrosomia have mainly focused on the risk factors and maternal outcome. Little attention has been given to the macrosomic infant even though they have high perinatal mortality and morbidity rates, particularly in our setting. Hence a need was felt to conduct this study on fetal macrosomia so as to aid in future identification of these pregnancies, anticipate their complications and plan on appropriate mode of management.

## Methods

This was a prospective matched case-control study conducted at MNH maternity and neonatal wards in Dar es Salaam, Tanzania. All infants with birth weight of 4000 g or greater delivered at the labor ward during the study period were selected as cases. The next infant of the same sex delivered with birth weight ranging from 2500 to 3999 g was selected and served as control. Cases and controls were matched for sex.

Three trained nurse midwives at the labor ward (one nurse per shift) were selected and assisted in recruitment of study participants. Babies were recruited within the first hour after delivery and after written informed consent. Recruitment of patients and sample collection was done at any time of the day. On average two to three cases and controls were enrolled daily.

Assuming the percentage of exposure (BMI > 28 kg/m^2^) of 13.6 % in controls [8], a confidence interval of 95 %, a power of 90 %, an assumed odds ratio of 2.5 and coefficient of correlation between cases and controls of 0.2, the sample size was estimated using the Epi-Info version 6, being 163 controls and 163 cases. Demographic and medical characteristics of mother and baby were obtained from antenatal charts and clinical notes. Infants weight, length, and occipito-frontal circumference was measured and entered in a standardized form.

At recruitment, a drop of capillary blood was obtained by heel prick of the baby for random blood sugar estimation. This screening was repeated during the second, fourth and sixth hour after delivery. Results were confirmed by checking plasma glucose levels. Blood was drawn for hematocrit, plasma calcium and glucose estimation. Infants suspected to have a fracture underwent X-ray examination for confirmation. They were reported by an experienced radiologist. Management was based on the standard treatment guidelines of the unit and all recruited babies were followed-up while in the ward until recovery and discharge or death. Discharge was sub-classified as discharge with/without disability.

Analysis was done using SPSS (Statistical Package for Social Science) software version 15. Means were compared by using Wilcoxon signed ranks test. Univariate analysis of risk factors was carried out using conditional logistic regression in order to determine strength of association with the outcome. Multivariate conditional logistic regression analysis was carried out on variables that were significant in the univariate analysis. Indications for caesarean section (C/S) and maternal and neonatal complications of fetal macrosomia were analyzed using binary logistic regression. Odds ratio with 95 % confidence intervals (CIs) were calculated for risk factors and complications. A *p*-value of less than 0.05 was considered as statistically significant.

## Results

During the study period October 2009 to March 2010, there were a total of 4528 deliveries of which 103 had a weight greater than or equal to 4000 g. The prevalence of fetal macrosomia was 2.3 %.

### Maternal and fetal characteristics

In the macrosomia group, mean maternal age was 29.9 years and was not significantly higher than the control group (*p*-value 0.05). Mean parity was significantly greater in the macrosomia group compared to the controls (2.4 vs. 1.2, *p*-value <0.001). Other parameters including weight at delivery, mean height and gestational age at delivery were also significantly higher among the cases compared to the controls. Mean birth weight in the macrosomia group was similar among male and female macrosomic neonates (*p*-value 0.3). Infants delivered by caesarean section had a greater mean birth weight compared to those delivered vaginally (4800 vs. 4200 kg). However this was not statistically significant (*p*-value 0.4). There were more males than females in the study population and the overall male to female ratio was 1.5:1 (Table 1).

**Table 1:** Maternal and fetal characteristics

	Macrosomia Mean ± SD	Controls Mean ± SD	*p*-value
Age (yrs)	29.99 ± 5.61	28.45 ± 5.97	0.057
Parity	2.43 ± 1.8	1.22 ± 1.5	0.000
Height (m)	161.5 ± 5.59	159.7 ± 4.98	0.02
Weight at delivery (kg)	83.8 ± 13.14	73.4 ± 11.87	0.000
Gestational age (wks)	38.7 ± 1.37	38.2 ± 1.16	0.005
Birth weight (g)	4.2 ± 0.31	3.2 ± 0.35	0.000
Males	4.3 ± 0.33	3.2 ± 0.36	
Females	4.2 ± 0.28	3.1 ± 0.35	
Birth weight by mode of delivery			
Caesarean section	4.3 ± 0.32	3.2 ± 0.37	
Vaginal delivery	4.2 ± 0.29	3.1 ± 0.36	
Serum calcium (mmol/L)	2.336 ± 0.15	2.334 ± 0.1882	0.93
Venous hematocrit (%)	54.9 ± 6.28	50.1 ± 5.30	0.000

### Maternal risk factors for fetal macrosomia

More than half of the mothers who delivered macrosomic infants had weight greater than 80 kg at delivery. In comparison, 73.6 % of women who delivered normal birth weight infants had a delivery weight less than 80 kg. Women with delivery weight greater than or equal to 80 kg were four times more likely to deliver macrosomic babies compared to controls (95 % CI [2.2–9.1]).

Majority of mothers delivering macrosomic babies were in the age group from 30 to 39 years (55.3 %); age groups less than 30 years and greater than or equal to 40 years were not significantly associated with macrosomia. Multiparity was present in 89.3 % of mothers in the macrosomia group. A significantly greater percentage (46.7 %) of mothers in the macrosomia group had previous history of delivering macrosomic infants (compared with 12.6 % among controls). Mothers having diabetes mellitus were 10 times more likely to deliver a macrosomic baby compared to those without diabetes mellitus.

Most of the deliveries in the cases and control groups occurred at gestational ages of 37–39 weeks (66.0 and 83.5 % respectively). However, macrosomic infants were four times more likely than controls to be delivered at gestational age greater than or equal to 40 weeks. None of the mothers in our study had pregnancy that lasted longer than 42 weeks.

When variables significant on univariate analysis were included in the multivariate logistic regression model, history of previous macrosomia and weight greater than 80 kg at delivery were the only maternal characteristics that were significantly associated with increased odds for macrosomic births (OR - 2.8 [1.1–7.2] and 2.5 [1.1–5.9], respectively (Table 2).

**Table 2:** Analysis of maternal risk factors for fetal macrosomia

	Macrosomia n (%)	Controls n (%)	Univariate analysis		Multivariate analysis	
			Odds ratio [95 % CI]	*p*-value	Odds ratio [95 % CI]	*p*-value
Weight at delivery						
< 80	45 (43.7)	76 (73.7)		1^a^		
80–89 kg	30 (29.1)	15 (14.6)	2.4 [1.2–4.8]	0.02	2.5 [1.1–5.9]	0.036
> 90 kg	28 (27.2)	12 (11.6)	2.8 [1.3–5.9]	0.009		
Height						
≤ 160	42 (40.8)	56 (54.4)				
> 160 cm	61 (59.2)	47 (45.6)	1.8 [1.0–3.3]	0.047		
Age						
< 30 years	44 (42.7)	58 (56.3)				
30–39 years	57 (55.3)	39 (37.9)	2.1 [1.2–3.7]	0.02	1.4 [0.6–2.9]	0.41
> 40 years	2 (1.9)	6 (5.8)	0.3 [0.7–1.6]	0.18		
Parity						
0–1	11 (10.7)	46 (44.7)		1 ^a^		
Para 2–4	76 (73.8)	53 (51.5)	2.0 [1.1–3.7]	0.03	1.7 [0.7–4.4]	0.24
Para ≥5	16 (15.5)	4 (3.9)	4.4 [1.3–11.9]	0.01	2.6 [0.5–13.1]	0.24
Diabetes mellitus						
Yes	12 (11.6)	3 (2.9)	10.0 [1.3–78.1]	0.03	4.2 [0.5–38.1]	0.20
No	91 (88.3)	100 (97.1)		1 ^a^		
Previous macrosomia						
Yes	48 (46.7)	13 (12.6)	6.3 [2.8–13.9]	<0.001	2.8 [1.1–7.2]	0.031
No	46 (44.6)	43 (41.7)		1 ^a^		
Gestational age						
37–39	71 (68.9)	93 (90.2)	0.4 [0.2–0.7]	1 ^a^		
40–42	32 (31.1)	10 (9.7)	4.1 [1.8–9.5]	0.001	1.9 [0.7–5.5]	0.21

^a^reference category

### Mode of delivery

The overall rate of C/S was 55.3 %. Other modes of delivery included spontaneous vaginal delivery (SVD), assisted breech delivery (ABD) and low cavity vacuum extraction (LCVE). There was no significant difference in these modes of deliveries between the two groups. Majority of the macrosomic infants were delivered by C/S was (61.1 %) followed by SVD (34.0 %). The rate of C/S was higher among cases compared to their controls (61.1 vs. 49.5 %) but the difference was not statistically significant (*p*-value 0.09). A greater percentage of macrosomic infants were delivered by emergency C/S compared to controls, but the difference was not statistically significant (Table 3).

**Table 3:** Mode of delivery

	Macrosomia N (%)	Controls N (%)	Total N (%)	*p*-value
Vaginal delivery	40 (38.9)	52 (50.5)	92 (44.6)	
SVD	35 (34.0)	48 (46.6)	83 (40.3)	0.52
ABD	4 (3.9)	3 (2.9)	7 (3.4)	
LCVE	1 (1.0)	1 (1.0)	2 (1.0)	
C/S	63 (61.1)	51 (49.5)	114 (55.3)	0.09*
Emergency	35 (34.0)	25 (24.3)	60 (29.1)	0.48
Elective	28 (27.2)	26 (25.2)	54 (26.2)	1
Total	103 (50)	103 (50)	206 (100)	

*p-value calculated to determine difference between caesarean and vaginal deliveries

### Indications for caesarean section

The commonest causes for caesarean deliveries included previous C/S scar (35.9 %), obstructed labor (29.8 %) and fetal distress (19.3 %). Some patients had more than one indication for C/S. Fetal macrosomia was identified as an indication for C/S in 20.6 % of cases, and this was significantly greater compared to the controls (*p*-value 0.003). There was no significant difference in other indications for C/S between the two groups (Table 4).

**Table 4:** Indications for caesarean section

	Macrosomia	Controls	Total N (%)	*p*-value
Fetal macrosomia	13 (20.6)	1 (1.9)	14 (12.3)	0.003
Fetal distress	11 (17.5)	11 (21.5)	22 (19.3)	0.7
Obstructed labor	22 (34.9)	12 (23.5)	34 (29.8)	0.3
Previous C/S scar	19 (30.2)	22 (43.1)	41 (35.9)	0.2
Uterine rupture	2 (3.2)	0	2 (1.7)	
Cephalo-pelvic disproportion	6 (9.5)	5 (9.8)	11 (9.6)	0.07
Total	63 (55.3)	51 (44.7)	114 (100)	

### Maternal complications

In the study population, 56.3 % of mothers in the macrosomic group developed at least one complication. In contrast, only 38.8 % of mothers in the control group developed any complication (p <0.05). Commonest complications in mothers delivering macrosomic infants included prolonged labor (27.2 %), 2nd degree perineal tears (22.3 %) and post-partum hemorrhage (PPH) (17.5 %). Mothers delivering macrosomic infants were five times more likely than the controls to develop PPH. Uterine atony, perineal tears and uterine rupture were the main causes of PPH. There were 3 cases of shoulder dystocia in the macrosomia group and uterine rupture occurred in 2 mothers in the macrosomia group (1.9 %). There was one maternal death in the macrosomia group; the cause of death was uterine rupture and PPH. No death occurred in the control group (Table 5).

**Table 5:** Maternal complications

	Macrosomia N (%)	Controls N (%)	*p*-value	Odds ratio [95 % CI]
Post partum hemorrhage (PPH)				
Yes	18 (17.5)	4 (3.9)	0.0015	5.24 [1.71, 16.09]
No	85 (82.5)	99 (96.1)		1
Prolonged labor				
Yes	28 (27.2)	14 (13.6)	0.02	2.37 [1.17, 4.83]
No	75 (72.8)	89 (86.4)		1
Uterine rupture				
Yes	2 (1.9)	0 (0.0)		
No	101 (98.1)	103 (100)		
2nd degree tear				
Yes	23 (22.3)	6 (5.8)	0.001	4.65 [1.80, 11.97]
No	80 (77.7)	97 (94.2)		1
Shoulder dystocia				
Yes	3 (2.9)	0 (0.0)		
No	100 (97.1)	103 (100)		
Maternal death				
Yes	1 (1.0)	0 (0.0)		
No	102 (99.0)	103 (100)		

### Neonatal complications

Neonatal complications occurred more frequently among the macrosomic group (44.3 %) compared to the controls (*p* < 0.05). The commonest neonatal complications among the macrosomic group were hypoglycemia (22.7 %), respiratory distress (16.5 %), birth asphyxia (14.4 %) and birth trauma (14.4 %). Mortality was significantly higher in the macrosomia group compared to controls (*p* <0.05). Main causes of death among the cases included stillbirth (6 cases and 1 control) and birth asphyxia. Out of the 12 macrosomic neonates who died, three were born to diabetic mothers. The commonest injuries were cephalhematoma and fractures (5.7 %) followed by nerve palsy (3.1 %). They were more likely to occur in infants delivered by SVD, ABD and LCVE. Hypoglycemia occurred more frequently in the macrosomic group compared to the controls (22.7 vs. 6.9 %). In 96 % of cases, hypoglycemia occurred within 6 h of delivery. Hypoglycemia was also more frequent in infants born to mothers without diabetes mellitus (23.5 %) compared to those born to diabetic mothers (16.6 %); however the difference was not significant (*p*-value 0.7) (Table 6).

**Table 6:** Neonatal complications

	Macrosomia N (%)	Controls N (%)	*p*-value	Odds ratio [95 % CI]
Birth asphyxia				
Yes	14 (14.4)	3 (3.0)	0.004	5.51 [1.53, 19.83]
No	83 (85.6)	98 (97.0)		1
Respiratory distress				
Yes	16 (16.5)	6 (5.9)	0.03	3.13 [1.17, 8.37]
No	81 (83.5)	95 (94.1)		1
Hypoglycemia				
Yes	22 (22.7)	7 (6.9)	0.002	3.94 [1.60, 9.72]
No	75 (77.3)	94 (93.1)		1
Hypocalcemia				
Yes	1 (1.0)	1 (1.0)	0.7	
No	96 (99.0)	100 (99.0)		
Polycythemia				
Yes	3 (3.1)	0 (0.0)	0.1	
No	94 (96.9)	101 (100)		
Death				
Yes	10 (9.7)	2 (1.9)	0.03	5.4 [1.16, 25.43]
No	93 (90.3)	101 (98.1)		1
Birth trauma				
Yes	14 (14.4)	1 (1.0)	0.0003	16.87 [2.17, 130.97]
No	83 (85.6)	100 (99.0)		1

### Neonatal complications of macrosomic babies by mode of delivery

A greater proportion of neonates delivered vaginally compared to those delivered by C/S died (12.5 vs. 7.9 %); however this difference was not significant.

Other complications such as hypoglycemia, respiratory distress and birth asphyxia occurred more frequently in the caesarean section group compared to those delivered vaginally. Compared to those delivered vaginally, a significantly higher percentage of neonates in the caesarean group developed respiratory distress. None of the neonates delivered by C/S sustained birth trauma whereas 35 % of infants delivered vaginally sustained birth trauma (*p*-value <0.001) (Table 7).

**Table 7:** Neonatal complications of macrosomic babies by mode of delivery

	Cesarean section N (%)	Vaginal delivery N (%)	Total N (%)	*p*-value
Death				
Yes	5 (7.9)	5 (12.5)	10 (97.1)	0.44
No	58 (92.1)	35 (87.5)	93 (90.3)	
Hypoglycemia				
Yes	16 (25.4)	6 (15)	22 (21.4)	0.2
No	47 (74.6)	34 (77.5)	81 (78.6)	
Birth trauma				
Yes	0	14 (35)	14 (13.5)	0.0001
No	63 (100)	26 (65)	89 (86.4)	
Birth asphyxia				
Yes	10 (15.9)	4 (10)	14 (13.6)	0.39
No	53 (84.1)	36 (90)	89 (86.4)	
Respiratory distress				
Yes	15 (23.8)	1 (2.5)	16 (15.5)	0.0003
No	48 (80)	39 (97.5)	87 (84.5)	
Total	63 (61.2)	40 (38.8)	103 (100)	

## Discussion

The prevalence of fetal macrosomia in our study was 2.3 %. Other studies in Africa have reported prevalence of 3.4 % in South Africa [17] and 3.5 % in Nigeria [18].

This low prevalence may be explained by the lower pre-pregnancy weight and low socio-economic status in our population.

### Maternal risk factors

There was a strong association between fetal macrosomia and maternal age greater than 30 years. This finding was similar to that seen in South Africa by Essel et al. [17]. This may be due to the fact that increasing maternal age may have an effect on maternal metabolism thereby increasing the growth velocity in the fetus. Pregnancy duration greater than 40 weeks was also significantly associated with fetal macrosomia. This finding parallels that observed by Cheng et al. [12]. Previous history of macrosomia was significantly associated with recurrence. A similar finding was noted in other studies [3, 7, 17, 18, 28]. This effect remains even after controlling for derangement in glucose metabolism [29]. Recurrence of fetal macrosomia may be due to greater maternal BMI at the time of conception, excessive weight gain between pregnancies as well as weight gain during pregnancy [30].

A history of diabetes mellitus (pre-existing or gestational) occurred more frequently among the cases compared to the controls. The finding of 11.6 % was much higher compared to 7.1 % in seen in Ibadan, Nigeria [18]. Diabetes in this group may be associated with obesity and thus greater insulin resistance, resulting in increased glucose availability to the fetus. Moreover, even in mothers without gestational diabetes mellitus (GDM) minor abnormalities in glucose metabolism during early and late gestation has been shown to increase the risk of fetal overgrowth [29]. Multiparity was present in 89.3 % of mothers delivering macrosomic infant. The finding was in keeping with other studies [8, 17]. It is speculated that increased parity associated with decreased insulin sensitivity results in greater amount of glucose being available for placental glucose transport and thus greater adipose tissue deposition in the fetus [31].

Mothers with a weight at delivery greater than or equal to 80 kg were more likely than their controls to deliver a macrosomic infant. Other authors have found that weight greater than or equal to 90 kg to be significantly associated with fetal macrosomia [17, 31]. Previous reports have shown that increased pre-pregnancy BMI as well as weight gain during pregnancy above the Institute of Medicine (IOM) guidelines to be associated with macrosomia [32]. It was not possible to determine whether this association existed in our population since majority of the mothers booked late during pregnancy and were not aware of their pre-pregnancy weight.

The high male to female ratio in the macrosomic group has been reported by other studies [7, 8, 18]. Ricart et al. showed that maternal glucose tolerance status was a significant predictor of fetal macrosomia in male but not in female neonates [33]. This may be explained by sexual dimorphism for insulin sensitivity, growth hormone-insulin growth factor 1 axis and cytokines. In addition, a prolongation of pregnancy could increase the exposure of the fetus to higher levels of glucose, insulin and different metabolic alterations if gestational diabetes is present. Catalano et al. also showed that average birth weight was significantly greater in males than females. This was attributed to increased neonatal fat free mass in males [31]. In our study, increased mean birth weight in males compared to females was not significant and could be due to chance.

### Maternal outcomes of fetal macrosomia

Postpartum hemorrhage, second degree perineal lacerations and prolonged labor were significant maternal complications in the macrosomia group. This was similar to findings noted by other authors [4, 8, 19]. The increased risk of PPH in this group may be due to perineal tears and prolonged labor resulting in uterine atony [28]. Moreover, uterine rupture occurred in two mothers delivering macrosomic infants and therefore been a cause of PPH in our study. There was one maternal death in the macrosomia group and this was due to uterine rupture and PPH. The maternal mortality rate of 971 per 100,000 was lower than that observed by Kamanu et al. [8]. The cause of death was however similar to that observed in their study and was due to an avoidable cause.

Sixty one percent of macrosomic babies were delivered by C/S in our study. This rate is four times higher compared to other studies [8, 19]. Although more infants were born by caesarean delivery in the macrosomia group, the difference was not significant compared to controls. Common causes of emergency C/S were obstructed labor and fetal distress and were not significantly different among cases and controls. This may be attributed by the fact that the study was conducted at a referral hospital and therefore a greater possibility of labor complications which necessitated emergency delivery by C/S in both groups. In our study, only 20.6 % of macrosomic infants delivered by C/S were identified by clinical examination. This highlights a need for identification of additional methods to identify macrosomic infants and determine appropriate mode of delivery prior to developing complications.

Shoulder dystocia occurred in 3 mothers delivering macrosomic infants and was associated with brachial plexus injuries. Similar to a study done by Iffy et al. [34] all the cases occurred with vaginal delivery. This suggests that shoulder dystocia is an important cause of birth related injuries particularly when macrosomic neonates are delivered vaginally and highlights a need of awareness and training of birth attendants on emergency management of shoulder dystocia.

### Neonatal outcomes of fetal macrosomia

Similar to other studies, macrosomia was shown to be associated with adverse neonatal outcomes [8, 21, 35]. Birth asphyxia was five times more likely in the study group compared to controls. Commonest causes were due to obstructed labor and fetal distress. Compared to infants delivered vaginally, a greater proportion of infants delivered by C/S had birth asphyxia. Delays in management of labor complications results in adverse pregnancy outcomes [36]. This emphasizes the need for continuous monitoring, identification of labor complications and avoiding delays in undertaking caesarean section so as to reduce complications such as birth asphyxia. Respiratory distress was also a significant neonatal complication among the macrosomia group and together with birth asphyxia was an important cause of admission to the neonatal ward. In this study, respiratory distress occurred more frequently in neonates delivered by C/S compared to those delivered vaginally. Das et al. found a similarly high frequency of respiratory distress in the macrosomia group which may be attributed to influence of increased caesarean deliveries and maternal diabetes on lung maturity [37]. Thus, the decision to opt for delivering macrosomic babies by C/S may have to be weighed against outcomes such as respiratory distress and increased admissions to the neonatal unit.

Birth trauma was greater in the macrosomia group compared to controls. Similar to other studies [8, 28, 34], the complication occurred only in neonates delivered vaginally. This finding suggests that complications such as birth trauma can be prevented by opting for C/S.

Hypoglycemia occurred in 22.7 % of macrosomic neonates. This was higher than that observed by Oral et al. [28]. Contrary to their findings, our study did not find any association of hypoglycemia with maternal diabetes status or birth weight greater than 4500 g. Though not significant, it is of concern that infants born to non-diabetic mothers developed hypoglycemia. This may be due to minor derangements in maternal glucose metabolism and therefore suggest that macrosomic infants require close monitoring for hypoglycemia regardless of maternal diabetic status. Not unexpectedly, most cases of hypoglycemia occurred in infants born by caesarean section. This may have been due to delay in initiation of feeding and intravenous dextrose in these neonates.

Similar to a study by Oral et al., there was no significant association between macrosomia with polycythemia and hypocalcemia [28]. All the cases that developed these outcomes were asymptomatic and therefore no treatment was given. Mortality was significantly associated with macrosomia as has been previously noted by other authors [3, 8]. Contrary to the study by Zhang et al. [21], most deaths occurred in macrosomic neonates in the weight range of 4000–4499 g compared to those weighing 4500 g. This may be due to differences in study design as well as differences in study population. Deaths due to birth asphyxia may have been preventable had the underlying problem been anticipated earlier and the mode of delivery been more planned.

### Study limitations

Due to non-availability of results of oral glucose tolerance test results during pregnancy some mothers with impaired glucose tolerance during pregnancy may have been missed. Diagnostic bias was minimized by measuring random blood sugar levels of all mothers in the study population. Only those with a previously documented history of diabetes mellitus or with abnormal random blood glucose readings were classified as having diabetes mellitus.

Majority of the mothers booked during the second or third trimester of pregnancy and it was not possible to measure the total weight gain during pregnancy. As a result, only weight measured at time of delivery was recorded.

Sixty three percent of the total sample size was reached and as a result reduced the power of the study to 80 %. This power is within acceptable value given the time limitations. Increasing the number of controls per case could have been used to increase the study power.

## Conclusion

The prevalence of fetal macrosomia at MNH was 2.3 % and an important cause of maternal and neonatal morbidity. Maternal risk factors include multiparity, previous history of macrosomia, presence of diabetes mellitus, gestational age of 40 weeks and above, delivery weight greater than or equal to 80 kg and maternal age ranging between 30 and 39 years. Complications included PPH, 2nd degree perineal lacerations, uterine rupture, and prolonged labor and maternal death. The neonatal complications included birth asphyxia, respiratory distress, hypoglycemia and death. We suggest that mothers should be screened using risk factors identified and be treated as a high risk delivery, with planned delivery and after care.

## Abbreviations

ABD: Assisted breech delivery
BMI: Body mass index
C/S: Caesarean section
GDM: Gestational diabetes mellitus
IOM: Institute of Medicine
LCVE: Low cavity vacuum extraction
MNH: Muhimbili National Hospital
MUHAS: Muhimbili University of Health and Allied Sciences
SVD: Spontaneous vertex delivery
